# Real-Time Seismic Intensity Measurements Prediction for Earthquake Early Warning: A Systematic Literature Review

**DOI:** 10.3390/s23115052

**Published:** 2023-05-25

**Authors:** Zhenpeng Cheng, Chaoyong Peng, Meirong Chen

**Affiliations:** 1 Institute of Geophysics, China Earthquake Administration, Beijing 100081, China; chengzp@cea-igp.ac.cn (Z.C.); chenmeirong@cea-igp.ac.cn (M.C.); 2 Key Laboratory of Earthquake Source Physics, China Earthquake Administration, Beijing 100081, China

**Keywords:** earthquake early warning, intensity measurements, point source model, finite fault, wavefield

## Abstract

With the gradual development of and improvement in earthquake early warning systems (EEWS), more accurate real-time seismic intensity measurements (IMs) methods are needed to assess the impact range of earthquake intensities. Although traditional point source warning systems have made some progress in terms of predicting earthquake source parameters, they are still inadequate at assessing the accuracy of IMs predictions. In this paper, we aim to explore the current state of the field by reviewing real-time seismic IMs methods. First, we analyze different views on the ultimate earthquake magnitude and rupture initiation behavior. Then, we summarize the progress of IMs predictions as they relate to regional and field warnings. The applications of finite faults and simulated seismic wave fields in IMs predictions are analyzed. Finally, the methods used to evaluate IMs are discussed in terms of the accuracy of the IMs measured by different algorithms and the cost of alerts. The trend of IMs prediction methods in real time is diversified, and the integration of various types of warning algorithms and of various configurations of seismic station equipment in an integrated earthquake warning network is an important development trend for future EEWS construction.

## 1. Introduction

Seismic intensity measurements (IMs) serve as indicators of the spatiotemporal variation in seismic intensity when an earthquake occurs. Seismic networks are used to detect earthquakes and quickly estimate the source location and magnitude at the data center (e.g., administration centers, local government buildings and national institutes). Subsequently, based on the estimated source parameters, the ground motion model (GMM) is used to calculate the IMs field to describe the distribution of the IMs field, which is centered at the epicenter. After an earthquake occurs, the real-time estimation of the IMs field enables individuals to rapidly assess the impact of the earthquake, and the intensity of the impacted area can be categorized according to the IMs strength. Individuals who receive warning information can evaluate the potential severity of the consequences of their decisions according to the intensity of the earthquake at their location, and thereby, they can implement more effective measures. This also furnishes a crucial framework for subsequent rescue operations and disaster evaluation.

With the development of earthquake warning technologies and methods, scholars have gradually deepened their understanding of earthquakes and have refined earthquake early warning systems (EEWSs), which has led to better earthquake warnings. This has resulted in operational EEWSs around the world that have been tested or implemented [1,2,3]. The construction of EEWSs can be traced back to the 1960s when the world’s first EEWS for railroad warnings, called UrEDAS, was built in Japan [4]. Subsequently, the SASMEX in Mexico was the first EEWS in the world to provide alerts to the public [5]. Then, in 2007, the first nationwide EEWS was implemented in Japan [6]. Ten years after Japan validated the importance of EEWSs, the regional EEWS in Taiwan and the KEEWS in South Korea started to issue warnings to the public [7,8]. ShakeAlert in the United States was also launched in 2021 to monitor earthquakes in the US West Coast [9]. EEWSs in countries such as Romania, Turkey and India can provide warnings to some users [10,11,12]. EEWS projects in mainland China, Greece and other countries are in the testing phase [13,14].

EEWSs can be mainly classified into two types of warning systems based on the alert methods used: regional warning systems and on-site warning systems. In the network-based EEWSs, the regional warning occurs after the basic earthquake parameters are obtained from the initial few-second *P* wave information from the near-source stations, and then the distribution of the IMs at different source distances is obtained by using the GMM, and this information is used to alert individuals of the potential damage zone of the earthquake. The regional warning system relies on abundant station information to provide a more accurate estimate of the earthquake source (ES) parameters. However, this system typically requires a longer processing time and cannot provide timely warnings in areas close to the epicenter. Conversely, the on-site warning system uses the initial part of the *P* wave waveforms observed by a single or few adjacent stations to predict subsequent IMs values at the same site. Therefore, the on-site warning system is commonly deployed for critical targets, with surrounding stations used to predict the intensity of the impending seismic waves. Due to its straightforward deployment, it can provide early warnings to the area around the epicenter [15].

The core infrastructure of a regional EEWS can be categorized into four modules: earthquake event detection and localization, estimation of magnitude, forecast of peak ground motion (GM) at the target location and issuing alert notifications [16]. Among these, the real-time prediction of the IMs can allow local inhabitants to promptly respond to the earthquake intensity that they may experience. Based on the IMs field and the division of the intensity distribution, the high-risk areas can be more appropriately identified. However, for strong and large earthquakes with fault scales up to hundreds of kilometers, the conventional point source EEWS that predicts magnitude from *P* wave information in the initial few seconds has some drawbacks. Large earthquakes can cause saturation problems with magnitude estimates. Moreover, the GM along the direction of the fault rupture (FR) will be stronger. The longer the FR, the stronger the GM at shorter distances from the fault. Regarding the 2011 *M* 9.0 earthquake off the Pacific coast of Tohoku, the predicted earthquake intensity in the Kanto area of Tokyo was lower than the actual observed intensity. The reason for this may be that the amplitude–magnitude saturation of seismic events with *M*w > 8 that reach the upper limit of seismometer measurements cause the magnitude to be underestimated. In addition, the inversion of the ES process indicated that the fault extended into the offshore Ibaraki Prefecture near Kanto, which led to an underprediction of the intensity [17,18]. This event accelerated the monitoring of offshore areas in Japan and the formation of a well-established offshore detection network, MOWLAS [19]. It also had a profound impact on the improvement and diversification of global EEWSs. For example, the length and width of ruptures were taken into account to obtain more accurate IMs predictions [20,21]. Various advanced neural network algorithms were used to improve the efficiency of early warning systems [22,23,24].

In this paper, we will focus on the real-time prediction methods of IMs for EEWSs in recent years and track the latest advancements. Firstly, we will investigate whether the ultimate magnitude scale can be predicted based on the initial rupture behavior of the earthquake, which is in line with various theories supported by EEWS methods. We then summarize the progress of regional and on-site warnings that are based on IMs predictions. The methods developed in the last decade are then summarized in terms of finite faults (FF) and simulated seismic wave fields, and the development and characteristics of these different algorithms are described in detail. Finally, the accuracy and timeliness of different algorithms are discussed, and user tolerance and warning cost are considered to evaluate the EEWS algorithm performance.

## 2. Theoretical Study on the Evolution of Earthquake Rupture

The question of whether large and small earthquakes exhibit different characteristics during early rupture is central to understanding the evolution of rupture over time. For point source EEWSs that rely on information from the first few seconds of source rupture to rapidly estimate the source parameters, the ability to quickly and accurately calculate magnitude is crucial. In the past decade, researchers have made efforts to investigate whether the initial rupture is deterministic, but the results remain inconclusive. Table 1 contains three different types of views on whether the initial rupture determines the final magnitude: deterministic assumptions, weak deterministic assumptions and no correlation assumptions, as well as the main discussion points of the different arguments supporting each view.

The concept of deterministic assumption refers to the variation in the final magnitude of earthquakes due to different nucleation processes, and the waveform of the initial few seconds can predict the final magnitude. Some scholars have held that the frequency or amplitude of the initial *P* wave is proportional to the final magnitude of the earthquake, which allows for an estimate of the magnitude before the completion of the rupture process [25,35]. Colombelli et al. [27] discovered that the peak displacement of small earthquakes increases rapidly in the initial phase, while the peak displacement of large earthquakes grows slowly, which led to the conclusion that the *P* wave peak displacement evolves differently with time for different earthquakes in the early stages of the rupture process. On the other hand, Rydelek and Horiuchi [29] questioned the idea of an deterministic assumption, contending that the earthquake nucleation process is universal and independent of the final magnitude, and that the rupture process is ultimately unpredictable. Rupture unpredictability suggests that it is impossible to predict the final magnitude of an earthquake based on early rupture behavior alone. Moreover, Meier et al. [30] analyzed moderate-to-large earthquake events and found no evidence of differences between small and large earthquakes in terms of the onset of rupture. Trugman et al. [31] compiled oversize seismic datasets by measuring the peak ground displacement (*P*_d_) of a progressively longer time window and assuming a time-dependent saturation of the linear relationship between log10 (*P*_d_) and magnitude (*M*w). The results indicated a universal growth pattern in the evolution of the *P*_d_ with time after the initial rupture of the fault, which was inconsistent with the deterministic model of seismic rupture.

In recent years, the focus of earthquake research has shifted from deterministic rupture nucleation to a concept known as rupture weak determinism, which concerns the ability to infer the final earthquake size after the nucleation. Melgar and Hayes [32] proposed a weakly determined model of rupture evolution, in which they found that large earthquakes rupture by slip pulses with self-similarity. They analyzed the variation in the average seismic moment rate over time by constructing an average source–time function with 0.5 magnitude units within the magnitude range of *M*w 7.0 to 8.5. The results indicated that the average seismic moment rate was significantly different in the first 10 s, whereby it was much smaller than it was during the duration of the earthquake. This suggests that a self-similar slip pulse is formed soon after the rupture starts. So, purely deterministic rupture mechanisms may be ruled out. Nonetheless, in some cases, weak or probabilistic forms of deterministic assumption can still be observed through a detailed analysis of seismic or geodetic data. Goldberg et al. [33] used seismic and geodetic data to study early rupture behavior and concluded that it was insufficient to infer the final earthquake magnitude (EM) from the first few seconds of the initial rupture. However, accurate estimates were possible in the tens of seconds before rupture completion, which indicated a weak certainty. Meier et al. [34] observed plenty of shallow crustal seismic data records and showed that inferring the final EM from the initial rupture characteristics was impossible. The rupture characteristics of small and large earthquakes can be distinguished only when the rupture development reaches a certain degree. Hutchison et al. [36] suggested that when the earthquake length reaches 20% of the total length, the final length of the fault and the EM can be predicted with accuracy, provided that the earthquake slip is accurately known and the fault structural maturity is considered.

## 3. Network-Based Earthquake Early Warning

Most of the regional warning methods based on seismic networks use point source algorithms (PSAs), which treat an earthquake as a point source, calculate the source parameters of the earthquake and predict future IMs based on the GMM. The implicit assumption of these EM estimates that are based on *P* wave signals forms the basis of deterministic assumption. When an earthquake starts, most of the rupture sliding of the fault is limited to a small area near the ES. Seismic energy is released in a brief period, and small-to-moderate earthquake events align with this assumption. However, predicting the final magnitude is difficult when the rupture extends hundreds of kilometers across the fault before it is complete, as the time available to reach the *P* wave window to predict the final magnitude is limited. The EM estimate is likely not yet stabilized after the arrival of the *S* wave at the station. Furthermore, high-pass filtering is carried out for strong motion records to minimize long-period drift during velocity and displacement integration, which diminishes the low-frequency content in seismic records [37]. Hence, one drawback of using PSAs for regional warnings is that for earthquakes larger than *M*w 7.0, the EM prediction suffers from a saturation of the magnitude estimate [35]. Another disadvantage is the uncertainty of using the GMM to predict IMs. The GMM is grounded on a substantial amount of seismic event statistics and uses epicenter distance and magnitude to estimate IMs at varying distances. The calculated IMs values represent only the expected results of using the standard regression equation and are not indicative of the actual IMs values at the target location. Thus, even if the exact earthquake location and magnitude are known when the GMM has a substantial error or the EM is imprecise, the estimated IMs will be unreliable. So, PSAs face limitations in three main aspects: the uncertainty of the ES parameter estimation, the saturation of the EM estimation and the uncertainty of the IMs prediction. Improvements in the PSA should be implemented from these perspectives.

### 3.1. Source Estimation Method

When it comes to regional warnings, to predict the IMs parameters, the seismic magnitude needs to be calculated first. The traditional methods for EM estimation are mainly carried out by using amplitude algorithms, period algorithms, multiparameter combination methods and Bayesian-based methods. The amplitude algorithm estimates the magnitude according to the amplitude parameter within a few seconds of the initial *P* wave. Wu and Zhao [38] established the correlation between the source distance (*R*), magnitude (*M*) and peak displacement (*P*_d_) based on the *P*_d_ of the initial *P* wave in the first 3 s. The period algorithm was first proposed by Nakamura [39], who argued that the EM was proportional to the frequency of the seismic wave and that the EM could be derived from the characteristic period *τ*_p_ of the seismic wave. While the single parameter-based magnitude estimation had a high uncertainty, the multiparameter combination method combined multiple parameters for magnitude estimation, which effectively improved the accuracy of the magnitude prediction. Huang et al. [40] found that the joint estimation of the two parameters *τ*_c_, *P*_d_ was more effective than the single *τ*_c_ method and could be used as a new EM estimation method. The virtual seismologist method proposed by Cua and Heaton [41] was an EM estimation method based on Bayesian conditional probability distribution theory. Real-time estimates and updates could be made based on the attenuation relationship between the observed IMs values, prior information and the distance from the earthquake epicenter. In addition, machine learning algorithms for magnitude estimation are also available. Mousavi et al. [42] used a deep learning approach to predict magnitude by directly extracting multiple *P* wave feature parameters from waveforms. Zhang et al. [43] proposed a fully convolutional neural network (CNN) model based on earthquake early rupture information, which could be used for real-time earthquake detection, earthquake localization and magnitude estimation, etc.

### 3.2. Ground Motion Model Based on M, R, VS30 with Shakemap

GMMs are mathematical models that predict how the intensity of IMs varies with earthquake size, distance and other factors, and they play an important role in earthquake engineering and seismic damage assessments. When researching the attenuation models of IMs, models generally consider the effects of three aspects: source characteristics, propagation medium and site conditions. The source characteristics cover the magnitude, fault type and plate location of the earthquake; the propagation medium focuses on the geometric dispersion and energy dissipation and absorption of seismic waves, which are usually expressed by the epicenter distance and fault distance; and the site conditions focus on the influence of the site type such as bedrock or soil fields on the IMs. In areas with abundant earthquake observational records, attenuation relationships are empirical formulas obtained from statistically analyzing strong motion observation records. A very well-known GMMs project is the Next Generation Attenuation (NGA) project led by the Pacific Earthquake Engineering Research Center (PEER), which shows the trends of the next generation of attenuation relationships in the global digital network with abundant strong motion data [44]. The first phase (NGA-West) started in 2003 and ended in 2008 and focused on the attenuation patterns in California, the U.S.A. and other seismically active regions around the world. Five working groups developed five NGA models based on different research objectives and the selection of different databases [45]. The second phase of the NGA project started in 2010 and is divided into two parts, NGA-West2 and NGA East. NGA-West2 is a continuation of NGA-West and aims to improve the models of NGA-West, such as in terms of their directional effects, basin effects and topographic effects. NGA East focuses on areas of stable seismicity in Central and Eastern North America [46]. In addition, NGA-sub is a derivative of the NGA project, and this is a model of attenuation based on subduction zone seismic records [47,48]. The NGA project promotes the development of GMM research, which has led to significant progress.

### 3.3. Country-Specific Examples

Among the EEWSs currently in operation, the ShakeAlert warning system in the U.S. is a typical regional warning system. The ElarmS algorithms in the ShakeAlert system calculate the seismic position, size and other source parameters by using the limited number of parameters extracted from the first few seconds of the initial *P* wave observed by seismic stations. ElarmS estimates the EM by using data from at least the first four stations of the seismic wave arrival network; it takes the two parameters *τ*_c_ and *P*_d_ of the initial *P* wave and then predicts the IMs values by using the GMM. This information is later integrated and updated in real time to generate IM prediction maps [26,49]. The second-generation ElarmS-2 algorithm has been recorded and modularized, with improvements made to the station network configuration. Its processing speed reduced the alert time by 6 s, which resulted in an overall enhancement in the warning performance [50]. Since ElarmS uses the short-term average/long-term average (STA/LTA) method to trigger earthquakes, this triggering method is very sensitive and prone to generating false seismic events. The third generation of ElarmS-3 uses a new teleseismic filter and trigger filter to reduce false alarms. The teleseismic filter distinguishes teleseismic signals with a filter bank by using the fact that the high-frequency components are more attenuated and the low-frequency components are less attenuated during the long-distance propagation of the teleseismic seismic waves. The peak ground velocity (PGV) values of the narrow bandpass filter traces with nine frequency bands ranging from low to high frequencies are calculated to distinguish whether they are teleseismic, which is determined by assessing whether the frequencies are in a set range. The trigger filter implementation involves the use of a series of algorithms to analyze the waveform characteristics of the seismic signal. First, an amplitude check is performed to determine whether *τ*_p_, *P*_d_, *P*_v_ and *P*_a_ are within the set range to exclude any abnormally small or large amplitude detriggers. A “range post-trigger” parameter (*R*) is introduced to ensure that the signal is not a single pulse or a rapidly shifted nonseismic signal. Finally, the horizontal-to-vertical amplitude ratio is checked to prevent *S* wave triggers from entering the system [51]. After calculating the source parameters using the ElarmS algorithm, another module in ShakeAlert, the earthquake information to ground motion (eqInfo2GM), can calculate the shakemap based on the source parameters and provide IMs information in a map or contour format. Therefore, users can select the appropriate alert method for their target location through the application, which allows them to focus on taking action based on local risks [52].

## 4. On-Site Warning Method of Earthquake Early Warning

The on-site warning system predicts IMs based on the characteristic parameters of the initial *P* wave observed by the seismic station. Compared with the regional warning method, the on-site warning method has fewer stations and has a relatively lower accuracy when estimating the source parameters. However, on-site warning only needs to predict future IMs values at the current location without considering the source parameters, which bypasses the uncertainty of source parameter estimation and the uncertainty of predicting the IMs field by using the GMM. This can provide a longer warning time for the area around the epicenter. Most of the current on-site warning systems use the *P*_d_ and *τ*_c_ of the initial *P* wave to estimate the IMs parameters. For example, the on-site algorithm in the ShakeAlert system provides warnings to locations up to 30 km away, and it provides 6 s of warning time to locations 50 km away [26]. The Italian SAVE on-site warning method has a success rate of over 80% in intensity prediction in the target area. The warning time is 8~10 s at 50 km and 15~18 s at 100 km [53].

### 4.1. P Wave Parameters

In addition to the *P*_d_ and *τ*_c_ mentioned above, other characteristic parameters of the *P* wave information, such as the squared velocity integral (IV2) and cumulative absolute velocity (CAV), can also be used for on-site warnings. Wurman et al. [54] used *P*_v_ and *P*_d_ to estimate the magnitude. Odaka et al. [55] proposed that the epicenter distance can be estimated by using the waveform envelope fitting parameter, and then they constructed an empirical magnitude–amplitude relation by using the *P* wave amplitude. Festa et al. [56] proposed a characteristic parameter IV2 related to the energy released by the earthquake and investigated the relationship between the initial radiated energy and the magnitude of the earthquake inferred from the IV2. Alcik et al. [57] investigated the relationship between the CAV and epicentral distance and magnitude and adopted an on-site warning method based on Peak Ground Acceleration (PGA) and CAV thresholds. Additionally, Wang et al. [58] proposed a method to estimate the earthquake magnitude in real time by using a displacement squared integral (ID2) for EEWSs.

### 4.2. Correlation between P Wave Warning Parameters and Ground Motion Model

Determining how to use the initial *P* wave warning parameters to quickly estimate the IM parameters is an important issue in on-site warning research. The GMM that considers *P* wave characteristic parameters and IM parameters is constructed in the on-site early warning system so that the earthquake warning information is issued in time when the set threshold value is reached.

The *P* wave amplitude, frequency, IV2 and other related parameters can be used with the IM parameters to construct a GMM. For each of the *P* wave warning parameters, the basic GMM can be expressed as

lgY = AlgX + B, (1)

**Figure 1:**
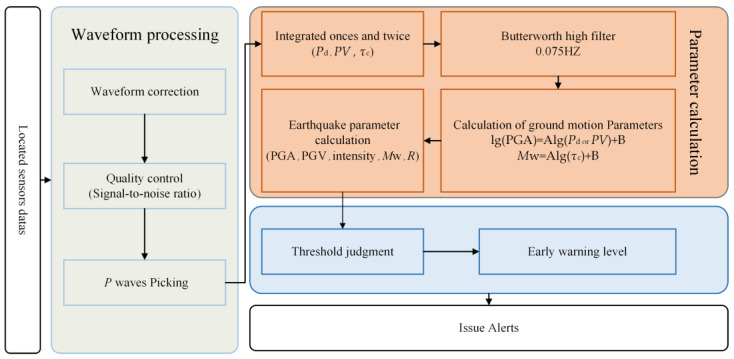
Threshold warning flowchart.

*M*w = Alg*τ*_c_ + B, (2)

where Y represents the PGV or PGA and X is the *P* wave parameters (e.g., *P*_a_, *P*_d_, *P*_v_). A and B are the coefficients to be obtained by the fit. For the threshold warning process, the implementation steps include (Figure 1):

- Waveform processing: when an earthquake is detected, remove the mean value and linear trend of the waveform and pick up the *P* waveform. Calculate the signal-to-noise ratio to eliminate data that may be contaminated by the noise for data quality control;
- *P* wave parameter calculation: integrate the accelerometer records once and twice to obtain the *P*_v_ and *P*_d_ records; filter them with a Butterworth high-pass filter with a cutoff frequency of 0.075 Hz to remove the low-frequency drift after the second integration; and obtain the *P*_d_, *P*_v_, *τ*_c_ and other parameters in the 3 s time window after the arrival of the *P* wave;
- Threshold setting: there is a good correlation between the seismic intensity parameter IMM and peak velocity and the early *P* wave peak displacement and IM parameter PGV [59]. By converting the intensity to the PGV, the threshold value of *P*_d_ is calculated by determining the empirical correlation between the *P*_d_ and PGV. Similarly, the threshold value of *τ*_c_ is determined by the correlation between *τ*_c_ and magnitude. For example, the *P*_d_ threshold and *τ*_c_ threshold are set to 0.2 cm and 0.6 s, respectively, for an earthquake with *M* > 6 and IMM ≥ 7 [15].
- Issue alert: judge whether the IM parameters exceed the set threshold, calculate the intensity level, determine the warning level and release the warning information.

Caruso et al. [53] employed a dataset of Italian earthquakes with magnitudes ranging from *M*w 3.8–6.0 to measure the *P*_d_ and *τ*_c_ after the arrival of the *P* wave. They established relationships between the *P*_d_ and PGV and *τ*_c_ and *M*w and derived connections between the *P*_d_, *τ*_c_ and epicentral distance (R). Using these parameters, the GM damage potential and range in the vicinity of the station could be quickly estimated. In contrast to seismic network monitoring, the threshold warning method utilizes single or multiple stations in proximity to the ES region for monitoring. An alert is promptly issued to the epicentral area upon detecting an earthquake that exceeds the predetermined threshold. This approach circumvents the uncertainties associated with predicting IMs by using the GMM. The practical applicability of the threshold warning method has been demonstrated worldwide, such as in China [60], Japan [61] and Italy [62]. All of these studies exhibited the accuracy and efficacy of this method. Wang et al. [63] analyzed the role of two early warning parameters, *τ*_c_ and *P*_d_, in magnitude estimation and proposed a threshold evolutionary magnitude estimation method based on *τ*_c_ and *P*_d_. They recommended utilizing *P*_d_ within a range of 10 km to predict events in the larger magnitude range and suggested the joint use of *τ*_c_ to reduce the underestimation error for larger earthquakes.

An additional approach to constructing GMMs is multiparameter joint prediction, which predicts IMs by making full use of *P* wave information. By extracting multiple *P* wave initial characteristics, we can establish more relationships between the *P* wave parameters and IM parameters or magnitude, which leads to more accurate IMs predictions [64]. Meier et al. [65] proposed a new amplitude estimation algorithm named Gutenberg that improves the accuracy of on-site warning systems by utilizing the broadband frequency information of seismic signals. By implementing a novel filter bank technique that measures the absolute peak amplitude of the *P* wave over time, joint Bayesian estimates of the EM, ES and station distance are produced. Additionally, the correlation between the initial *P* wave parameters and the IM parameters is explored. Peng et al. [66] used filters of different orders to establish further relationships between initial *P* wave parameters and IM parameters and thereby improved the accuracy of on-site warning systems. Wang and Zhao [67] investigated the initial *P* wave estimation of IMs using the Wenchuan database. They selected eight characteristic parameter attributes of the *P* wave in a 1 to 3 s time window and established relationships with four IM parameters, and then they subsequently analyzed the accuracy of their estimation.

### 4.3. Ground Motion Model Based on Artificial Intelligence Technology

Artificial intelligence techniques provide another new research idea for on-site warning systems; using one or two *P* wave warning parameters to construct a simple GMM for fitting is no longer necessary. Multiple *P* wave feature parameters can be extracted from the raw waveform data by using machine learning to construct an artificial-intelligence-based GMM to establish a more complex relationship between the feature parameters and the IMs. Hsu et al. [68] extracted some *P* wave feature parameters from the first few seconds of an earthquake at a single station and used support vector regression methods to build a regression model to predict the PGA based on these features. Song et al. [69] used the least squares support vector machine model to construct a continuous prediction model of the PGV by selecting seven characteristic parameter inputs of the *P* wave. Chiang et al. [24] proposed the use of intelligent strong motion prediction to predict IMs, whereby a CNN is used to determine the relationship between the features extracted from the initial *P* wave and the strong motion is used to predict whether the GPA of subsequent waves exceeds a predetermined threshold. To reduce the complexity of multiparameter calculations, Hsu et al. [70] selected the two parameters of the first 3 s of two *P* wave signals and used an artificial neural network algorithm with the introduction of different Site Characteristic Parameters for PGA prediction.

Moreover, artificial intelligence techniques are used to predict the IMs of the target location directly from the observed *P* wave information. Hsu and Huang [71] performed a multiscale analysis to estimate future IMs based on the observed *P* wave data of the first 3 s of an earthquake detected at a single station by using the CNN approach. Jozinović et al. [72] introduced a technique for predicting the intensity of an earthquake by using a deep CNN. Using the information contained in the first 10 s *P* wave of the stations of neighboring earthquakes, a CNN model was used to predict the intensity of earthquakes at more distant stations, which could provide an estimate of the IM within 15–20 s after the occurrence of an earthquake. Compared with the GMM method, the CNN outperformed the GMM in terms of the residuals of the data. The CNN required no ES parameters, the receiver was located at the epicenter and its uncertainty was lower than that of the GMM. However, the weak interpretability of machine learning models can lead to false positives or false negatives, which makes it difficult to assess incorrect predictions [73]. The dataset was selected from stations in a specific geographic area for training, and if other stations are added, the model has to be retrained. Although it performed well for the complete sample, poor data quality (e.g., data loss, station failure) can occur in reality, so data from real situations are needed for model training.

## 5. Intensity Measurements Estimation Based on Finite Fault Model

Treating the rupture as a point source in a large earthquake, the *P* wave frequency or amplitude in relation to the EM will saturate [74]. The FF algorithm aims to solve the point source saturation problem by estimating the rupture size. According to the rupture unpredictability theory, earthquake nucleation processes are similar and cannot be distinguished from the initial waveform [20]. Therefore, the FF algorithm focuses on the evolution of the seismic record over time and continuously updates the estimation of the magnitude until the end of the rupture. Moreover, the method of predicting IMs by using a GMM and the point sources algorithm underestimates the intensity at the target location [75]. The EM estimation and the use of the standard isotropic GMM consider only the epicenter distance and do not take into account the azimuthal variation in the seismic wave propagation. For near-fault locations of large earthquakes, the distance and azimuthal distribution of GM propagation have a finite rupture effect, so the length of the fault is not to be ignored. The directionality of the fault and the vertical distance from the rupture determines the GM at a given location. Understanding the geometric parameters of large earthquake faults is critical to ensure the accuracy of earthquake-intensity estimations.

### Intensity Measurements Estimation Based on Finite Fault Template Matching

Böse et al. [76] proposed the finite fault rupture detector (FinDer) algorithm to estimate the location, length and direction of rupture from the spatial distribution of the current observed high-frequency PGAs. When a large earthquake is monitored, some stations are the first to monitor the GM. FinDer performs spatial interpolations to obtain the interpolation matrix *b*(*x*, *y*) based on the maximum value of the GPA recorded by the dense seismic network. The interpolation matrix *b*(*x*, *y*) is classified for near and far stations to generate the binary image *f*(*x*, *y*). In the classification, if the PGA of the interpolated grid point is greater than the threshold GPAtr, the grid point is assigned a value of 1, whereas others are 0, as shown in Equation (3). Then, *f*(*x*, *y*) is matched with the near-field template to determine the best position of the template in the data:

(3) $$f\left(x,y\right)=\left\{\begin{array}{c} 1,\;\;\;if\;b\left(x,y\right)\geq {\mathrm{PGA}}_{\mathrm{tr}}\\ 0,\;\;\;\mathrm{Otherwise} \end{array}\right.$$

**Figure 2:**
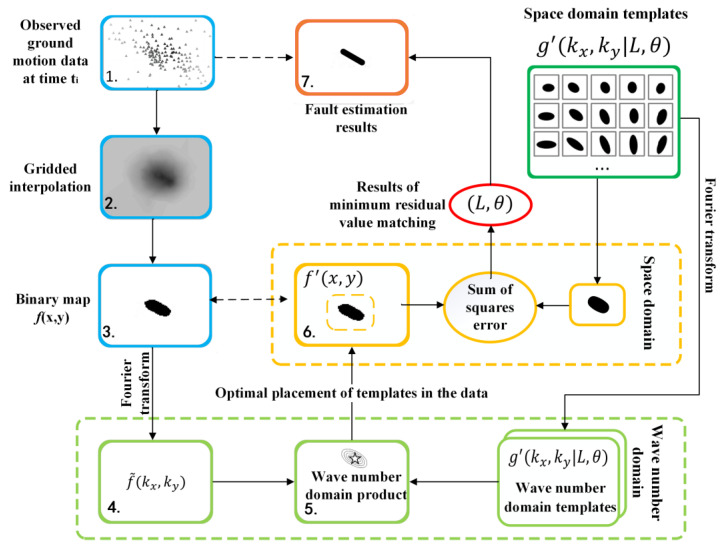
The FinDer method.

The theoretical near-field template is a graph of the near-field range formed by constructing different fault lengths that correspond to different fault distances under a given PGA threshold value according to the GMM. Using the attenuation relationship of the Joyner–Boore distance (*R*_jb_), a template library *g*(*x*, *y*|*L*, *θ*) between different fault distances and the corresponding PGA is established for large earthquakes above *M*w 6.0. Böse et al. [20] improved this attenuation relationship and proposed that the *R*_jb_ should be judged based on the EM and PGA thresholds, as in Equation (4):

(4) $$\log_{10}\left({\mathrm{PGA}}_{\mathrm{tr}}\right)=\left\{\begin{array}{c} 0.73Mw-7.2\times 10^{-4}\times \left[\sqrt{R^{2}+9}+C\left(Mw\right)\right]-\\ 1.48\log_{10}\left[\sqrt{R^{2}+9}+C\left(Mw\right)\right]-0.42 \end{array}\right\}+\log_{10}\left(1.1\right)$$

(5) $$C\left(Mw\right)=1.16\exp\left[0.96\times \left(Mw-5\right)\right]\times \left[\arctan\left(Mw-5\right)+\frac{\pi }{2}\right]$$

where *R* is the fault distance (km) and GPAtr is the PGA threshold (m/s^2^). The fault geometry parameters are estimated in real-time by matching the binary image *f*(*x*, *y*) with a set of near-field templates *g*(*x*, *y*|*L*, *θ*) in the wave number domain to find the minimum error and calculate the current position, length (*L*) and strike (*θ*) of the rupture (Figure 2).

FinDer quantifies the current degree of FR and is suitable for seismic networks with dense, evenly distributed stations and small station spacing. The smaller the spacing and the denser the distribution of the station network, the faster the detection of large earthquakes and the more accurate the estimation of fault parameters. However, in regions with sparse stations, there is a decrease in the accuracy of the parameter estimation [77]. Compared with the PSA, FinDer takes into account the fault distance from the target point and is more precise for the prediction of IMs. In a retrospective study whereby the FinDer algorithm was used for the Wenchuan earthquake, the alarm was triggered 12 s after the earthquake, and the final estimated rupture length and strike were consistent with the actual findings [78]. In the improved version of the FinDer algorithm, the applicability of FinDer is extended to the entire EM range using a list of PGA thresholds, which compensates for the previous generation FinDer algorithm, which was only applicable to large earthquakes [20]. Additionally, a group of asymmetric templates had been added to provide more exact rupture estimates along curved faults [79]. FinDer cannot predict future FR lengths, and it is used to identify faults where ruptures were occurring. Two extensions of FinDer, namely FinDerS and FinDerS+, were presented for this purpose, which could provide reliable and improved real-time IMs predictions [80]. However, after applying FinDer algorithms, it was found that the algorithms are limited by the structure of the station network. When earthquakes occur at the edges of the network or in areas outside the network, the magnitude and location of the earthquakes are usually misclassified. Although the FF algorithm is slower at providing warnings than the PSA, it can predict the earthquake intensity more accurately before ground shaking is felt, and it can make precise predictions of intensity over a larger area.

## 6. Intensity Measurements Prediction Based on Simulated Seismic Wave Fields

Simulated seismic wavefield-based methods can be used to predict future seismic wavefields directly from the current seismic wavefield. The future IM parameters (the PGA or PGV values) are predicted from the observed IM parameters by the seismic networks. For earthquakes with a long duration and large rupture extent, and for multiple simultaneous earthquakes, wavefield methods are well suited to solve problems regarding accuracy, whereas traditional methods for estimating ES parameters have limitations in these aspects. Hoshiba et al. [17] pointed out that with the 2011 *M*w 9.0 earthquake in Japan, the length and directionality of the FR were not considered, which resulted in the predicted intensity of the earthquake in the Kanto area being less than the observed intensity. Since the EEWS for point sources is based on the fact that the point source radiation is isotropic, as many stations from different directions and locations as possible are needed to accurately estimate the source parameters. When the FR length is large and strong along a certain direction, the accuracy of the magnitude estimate is reduced. Another factor is that after a large earthquake, the EEWS cannot distinguish between simultaneous aftershock sequences and fails to correctly estimate the source location and magnitude, which directly leads to an error in intensity prediction. In the wavefield-based approach, the actual current observations are reflected in the estimation of the current situation as much as possible. The difference between the estimated current situation and the actual current observations is minimized before making predictions [81]. Table 2 presents a literature review on wavefield simulations, which focuses on different approaches based on wavefield simulations.

### 6.1. Numerical Shake Prediction for EEWS

To address the shortcomings of the traditional way of predicting IMs by using the PSA, Hoshiba [82] proposed a new method of applying the Kirchhoff–Fresnel boundary integral equation to predict subsequent IM parameters directly by monitoring the current wave field by using a dense seismometer. This method does not require ES parameters and considers the rupture directionality, ES size and the effects of multiple simultaneous earthquake occurrences. However, it requires a dense network of stations to identify the wavefront spread and propagation, consider the effects of the amplification factor between the observation and target points and estimate the wave propagation direction in real time. Hoshiba and Aoki [83] conducted numerical shake predictions by using this method; namely, they used the data assimilation technique to determine the current distribution of the intensity parameters and then utilized the Kirchhoff–Fresnel integral to simulate the wave propagation instead of using radiative transfer theory. The data assimilation technique is an interpolation method that precisely estimates the current wavefield. The wavefield distribution at time t is predicted depending on the observed *t* − Δ*t* and the previous wavefield distribution. The real-time estimation of IMs is achieved by minimizing the difference between the prediction of the current situation and the actual current observation by comparing the observed wavefield at moment t with the wavefield predicted at moment t. Radiative transfer theory predicts future wave fields by simulating wave propagation without source parameters when the spatial and temporal distribution of the waves is known. Compared with the Kirchhoff–Fresnel integral theorem, it is less computationally intensive and more computationally time efficient. However, the method requires a dense observation network, and the accuracy of the wavefield estimation decreases when fewer stations are available. Ogiso et al. [84] improved the numerical ground shake prediction by adding a path term that can predict GM by using heterogeneous attenuation structures.

### 6.2. Intensity Measurements Prediction Based on Propagation of Local Undamped Motion

Kodera et al. [87] streamlined the method of Hoshiba [82] and proposed a simple wavefield estimation algorithm called the Propagation of Local Undamped Motion (PLUM) method. This method allows for continuous observations and the reception of station data and enables the real-time monitoring of target points. Studies on the 2011 *M*w 9.0 earthquake and the 2016 Kumamoto earthquake in Japan showed that the PLUM method delivered alerts faster than conventional methods and could provide early warnings for areas closer to the epicenter. Kodera et al. [21] discussed the principle of the PLUM method in detail and incorporated it into the JMA’s early warning system in 2018 to increase the accuracy of the EEWS. The performance evaluation of the PLUM method after one year of operation demonstrated improvements in the timeliness of the destructive earthquake alerts and a reduction in the number of missed alarms [88].

#### 6.2.1. Principle of PLUM Method

The PLUM method consists of two parts: GM prediction and event construction. The former predicts the seismic intensity of all target sites by using the PLUM method. The latter handles the release, identification and termination of seismic events depending on the observed real-time seismic intensities. For real-time GM predictions, the seismic intensity at the target site is given by Equation (6):

(6) $$I\left(r,t\right)\approx F_{0}+I\left(r_{1},t-\frac{\left|r-r_{1}\right|}{v_{0}}\cos\left(\theta^{\prime }-\theta \right)\right)$$

**Figure 3:**
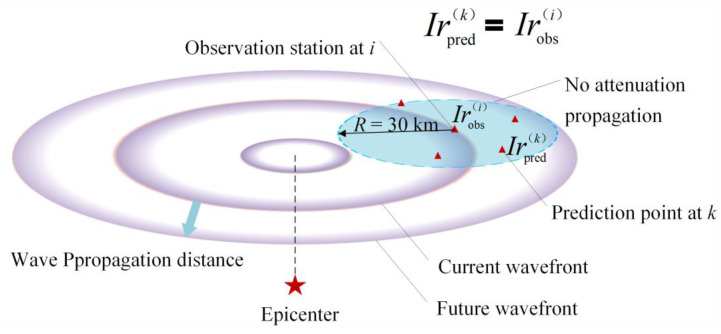
Schematic illustration of PLUM’s prediction algorithm.

Here, *I* (*r*, *t*) represents the seismic intensity, *r* is the location of the predicted target point, *r*_1_ is the location of the observation point, *v*_0_ is the velocity at *r*_1_ and *θ* is the propagation direction. *F*_0_ denotes the amplification factor. When the distance between the observation point and the prediction point is much smaller than its distance from the ES, the equation can be simplified to Equation (7), shown in Figure 3:

(7) $$I_{pred}^{\left(k\right)}=\underset{i\in C_{R}}{\max}\left\{Ir_{obs}^{\left(i\right)}-F_{0}^{\left(i\right)}\right\}+F_{0}^{\left(k\right)}$$

*i* and *k* represent the location, $I_{pred}^{\left(k\right)}$ is the predicted seismic intensity at *k*, ${Ir}_{obs}^{\left(i\right)}$ is the observed seismic intensity at location *i*, CR denotes the area centered at the prediction point with a circle of radius *R* and *F*_0_ is the magnification factor, which indicates the seismic intensity difference. The predicted seismic intensity at the target location is taken as the maximum value of the observed real-time seismic intensity in a circular area of radius *R*, and the site effect is also taken into account. When the radius *R* is small, the wave can propagate without attenuation in the region. The early time window (TW) can be defined as Equation (8):

(8) $$t=\left(\frac{R}{V_{s}}\right)-\Delta t_{SD}$$

*Vs* is the *S* wave velocity and Δ*t_SD_* is the system delay. To ensure the accuracy of the intensity prediction, the radius *R* is required to be small. In the ideal case, the system delay is considered to be zero. When the maximum GM propagates to the station, the TW that can be received at the edge station of radius *R* depends on the size of the *S* wave velocity and radius *R*. The maximum TW is about 10 s (*R* is 30 km and *S* wave velocity is 3 km/s).

#### 6.2.2. Improvement and Testing of the PLUM Method

The PLUM method must be employed when centered on a station, and it is used to warn individuals in an area with radius *R* of an earthquake. Therefore, the TW is short regardless of the distance from the source of the earthquake. To extend the TW, the radius (*R*) must be expanded. However, since waves demonstrate undamped propagation, if all the areas within the radius (*R*) are predicted to experience the maximum observed intensity, the expansion of the radius will increase the uncertainty of the intensity prediction and may overestimate the intensity. To solve the problem of overestimating the intensities of earthquakes around stations and to improve the accuracy and timeliness of the PLUM method for early warnings, an improved PLUM method was proposed by Kagawa [85] after analyzing the *M*w 6.6 magnitude earthquake that occurred in Tottori Prefecture, Japan, in 2016. This method improved the accuracy of the intensity estimation by introducing an attenuation factor in the wavefield. The maximum value of the intensity estimates at grid points within one kilometer of the earthquake was also considered for the comparison with the observed value.

Another way to improve the accuracy of the intensity estimation is to construct a suitable attenuation model based on the observations of the actual distance attenuation relationship between the current wavefield and predicted intensity. Kodera [86] developed a method that combines the GMM with PLUM by adding a wave attenuation model and the method of Approximation by Local Pseudohypocenter Attenuation (ALPHA) to improve the TW and range of the PLUM method. The ALPHA method sets the reference station that exceeds the threshold as the reference station and considers this station as a seismic source. It is assumed that the seismic wave propagates along the direction of the lowest seismic intensity, which corresponds to the direction of the maximum wavefield gradient. ALPHA is used to analyze the observed intensities of all stations within the radius (*R*) of the reference station and find the orientation of the station with the lowest intensity to obtain the wave attenuation direction. Using different types of local distance attenuation models, the most suitable GMM model is selected to predict the intensity within the range (*R*). The study showed that the PLUM method and ALPHA can complement each other and can provide a longer TW for near- and far-earthquake regions, respectively [86].

The advantages of the PLUM method are that the algorithm is simple, can accurately predict the intensity distribution from faults with large rupture scales, has a better reliability than the PSA for forecasting complex earthquake sequences, and does not underestimate the intensity of earthquakes. The PLUM algorithm combined with the conventional algorithm can improve the TW and make up for the insufficient prediction of the PSA. Cochran et al. [89] were the first to experiment with the applicability of the PLUM method in the California region. The California region is very different from Japan in many aspects, such as their station equipment, station spacing and intensity classification. The insufficient station density and the susceptibility-to-noise interference at low thresholds make it impossible to apply the PLUM method directly to the California region. For this reason, Cochran et al. [89] made some improvements to the PLUM method:

1. Grid definition: each station is connected to its six neighboring stations regardless of spacing. When a station monitors motion above the threshold, it sends its maximum predicted value to the surrounding six stations;
2. Intensity modification: the intensity $I_{JMA}$ is changed to $I_{MMI}$, and $I_{MMI}$ is calculated from the PGA and PGV;
3. Dual station triggering algorithm: when both adjacent stations trigger the threshold, the station whose maximum value of ground vibration is triggered satisfies the primary threshold, and the adjacent triggered station meets the secondary threshold to solve the noise-spike false-alarm problem.

The test results showed that the PLUM algorithm solved the problem of no alerts being sent in areas near the ES and could operate independently, which serves as a complement to the ShakeAlert system, and it also performed well when warning individuals of the California earthquake [90].

To solve the issues regarding the applicability of the PLUM method in different regions, further research is needed on the schemes and threshold methods that are suitable for local conditions. Minson et al. [90] explored the accuracy and timeliness of the PLUM method at different warning ranges at the county level, district level, and 50 km earthquake grid while using three different alerting strategies. Kilb et al. [91] used two seismic datasets to investigate the accuracy and timeliness of the PLUM method for earthquakes in the U.S. west coast. Cochran et al. [92] further investigated the optimal prediction radius and alert thresholds for the PLUM method and found that having a larger prediction radius and smaller intensity thresholds resulted in a better performance. For example, for a target MMI 4.5, the warning quality was higher when setting a prediction radius of 60 km and when MMI 4.5 was the threshold degree.

## 7. Intensity Measurements Evaluation Methodology

The goal of an EEWS is to provide information about IMs (e.g., the PGA, PGV, intensity) at the user’s location to mitigate earthquake damage. With EEWSs, the methods used to obtain IMs can be divided into two categories: one is calculating the source parameters by using the PSA and then obtaining the IMs distribution map by using the GMM, and the other method is based on FF or simulated seismic wave fields to provide the predicted IMs values in a region. Therefore, there are two methods used for the evaluation of IMs, point source assessments and IMs accuracy assessments [93]. Traditional point source parameter methods generally consider the timeliness of alerts, namely how quickly an alert can be issued since the origin time to indicate the timeliness of the algorithm, and they also consider the accuracy of ES estimations by comparing whether the predicted ES parameters match the actual seismic observations to characterize the accuracy of the algorithm [49,94]. The IMs accuracy evaluation method is used because different parameters are characterized by different algorithms; for example, the PSA generally uses ES parameters (e.g., information on magnitude, location and onset time), and FF algorithms calculate fault geometry parameters. An evaluation method is needed to compare the performance between different algorithms. From the user’s point of view, assessing the method based on the accuracy and timeliness of the prediction of the IM parameters is an objective evaluation method used to measure the performance of different algorithms [95].

### 7.1. Evaluation of Intensity Measurements Accuracy Based on Different Algorithms

The key parameters for the general evaluation method of the point source algorithm are magnitude, epicenter location, origin time and alarm time. To test the algorithm, historical earthquakes are retrospectively analyzed, and the predicted source parameters are compared to the true values in the earthquake catalog. The aim is to determine whether the errors regarding the magnitude and epicenter location are acceptable and whether there is sufficient warning time between the start time and when strong motion reaches the target location. For example, ShakeAlert’s point source code evaluation scheme sets five parameters, i.e., the goodness of the magnitude (Mg); goodness of the epicenter (Eg); goodness of the origin time (Og); goodness of the alert time (Tg); and a combination of Mg, Eg, Og and Tg into an alert assessment (Ag) to evaluate the performance of the point source algorithm [93].

Point source assessment methods are not applicable when using the FinDer method to determine the real-time inversion of FF lengths or when using the PLUM method to predict future GMs. However, the ultimate goal of all algorithms is to be able to accurately predict the intensity of the target location. However, an accurate IMs prediction requires not only the location and magnitude of the ES, but it also needs to have source faults, stress drops, path effects and local site characteristics taken into account. The accuracy assessment of IMs predictions can consider the effects of these factors and respond to the IMs prediction performance of the algorithm. By setting an IM parameter threshold, the method compares the IMs field distribution predicted by the algorithm with the real observed IMs field and triggers an alarm if the predicted IMs in the target area exceed this threshold. The “predicted” field is calculated by using the algorithm’s source parameters, and the observed field is derived from all available real observations of IMs. Finally, the alarms are classified into four categories depending on whether the alarm is triggered in the target area or not (Table 3). Meier [95] classified the accuracy of ideal PSAs and FF algorithms, whereby the FF algorithm had a true positive rate of 78% and thus outperformed the PSA. Meier et al. [96] analyzed the alarm performance of the PLUM, EPIC and FinDer algorithms by considering the precision of the alerts and the recall of the alerts. The results showed that all the algorithms had the best classification performance in the lowest threshold case. The classification accuracy of the PLUM algorithm was the highest for most threshold cases, followed by FinDer and finally EPIC.

The relationship between the TW and threshold is also important in the evaluation of the algorithm, where higher thresholds result in shorter TWs and lower thresholds result in more false alarms. Meier [95] explored the TW of the ideal PSA and FF algorithms and showed that at a specific threshold, the TW in the high-intensity region was short, i.e., no more than 8 s, and tended to occur in the epicenter region, while the TW could reach 1 min in the low-to-medium intensity regions. Minson et al. [97] discussed the timeliness of early warning systems and found that the TW was related to the threshold size and distance, whereby the farther the distance, the smaller the threshold set and the longer the TW. Moreover, at any distance, small thresholds can give alerts faster than large thresholds, and issuing alerts using low thresholds can extend the TW. This is related to the length of the earthquake FR. The greater the length of the rupture, the more energy is released and the greater the surface intensity. The threshold size cannot be determined until the rupture has stopped, and a longer time is required to identify the magnitude of the earthquake. Considering the tolerance of the user, the EEWS performs best when the threshold value for the alarm is set below the threshold value of the damage to the user. Although such thresholds lead to an increase in the number of false alarms, the number of missed alarms is effectively reduced, and the maximum benefit can be obtained [98]. However, low threshold alerts are not suitable for all scenarios. Minson et al. [99] studied a case example of an ideal warning system applied to a railroad system and showed that low threshold alerts, while having a longer TW, might not be applicable when the resulting unnecessary alerts cause too much damage. Appropriate thresholds and warning methods should be selected for specific application scenarios.

### 7.2. Impact of Alert Costs on the Intensity Measurements Accuracy

The accuracy assessment of IMs predictions can be considered in terms of the cost of alerts. The risk avoidance cost is incurred when an alert is issued, regardless of whether it is a correct alert or not. The EEWS is only meaningful if the cost of taking action is less than the potential cost of the losses. Additionally, the correctness of an alert is related to the setting of the intensity threshold. When the thresholds are high, the accuracy of the alerts is high, but high thresholds are not a good choice considering the need for the high stability of precision instruments and people’s perception of ground shaking. However, low thresholds can generate more unnecessary alarms and lead to higher alarm costs. To determine the optimal alerting strategy, Minson et al. [98] presented a normalized cost reduction metric in terms of the CR to describe the EEWS, as in Equation (9):

(9) $$CR=\frac{C_{without\;EEW}-C_{EEW}}{C_{without\;EEW}}\times 100\%=\frac{1-\frac{f+1}{r}}{m+1}\times 100\%$$

*m* is the ratio of the number of missed and correct alarms, *f* represents the ratio of the number of false and correct alarms and *r* is the degree to which users tolerate false alerts. The CR is the ratio between the cost of damage that could have been prevented by the user receiving the alert and the cost of loss caused by taking action. A higher CR represents a more effective warning system. A CR value of 1 means no loss, and a value less than 0 indicates that the cost of alerting exceeds the cost of possible losses. Kodera et al. [87] analyzed the FinDer, PLUM and EPIC algorithms with the CR ratio and found that the PLUM and FinDer algorithms usually obtained a higher CR than EPIC because they had fewer false negatives. In a cost-reduction framework, missed alerts are inherently worse than false alerts as the failure to mitigate is more costly than the unnecessary measures taken.

## 8. Discussion

Real-time IMs predictions are an important part of the EEWS implementation process. They can provide an effective TW for near-source areas as well as different types of intensity distribution maps such as the PGA and PGV. This information will help with the delineation of earthquake disaster areas, seismic hazard analyses and disaster relief guidance for early earthquake hazard mitigation. In the past, the early EEWS deployed only had the PSA and threshold warning methods, which could provide little information on the ES and limited the warning range to a small number of areas or important projects. Now, owing to the increasing construction of seismic networks, the methods and facilities of early warning systems are advancing. In the past decade, the FinDer algorithm and PLUM algorithm for FF have been tested in the United States, Japan and Europe and are considered to be promising, as they have achieved good results. These methods improve the richness of seismic information acquisition and increase the fault tolerance of seismic detection. Therefore, future EEWS construction system should be built with a variety of earthquake monitoring equipment and by using multiple EEWSs, which can collect earthquake information from different perspectives; as a result, the early warning information will be more abundant, timely and reliable. As part of the China Earthquake Early Warning and Intensity Velocity Project, an integrated infrastructure with velocity meters, accelerometers, intensity meters and GNSS stations will be built to complete the integration of different types of earthquake-monitoring networks. Multiple systems such as the integrated PSA, FF algorithm and predictions based on simulated seismic wave fields will be used to enrich the ES information and produce a more reliable early warning result [14]. The early warning information will be continuously updated and integrated with the increase in recorded station information to achieve the real-time monitoring of earthquakes.

To cope with seismic monitoring in remote areas and areas not covered by stations, building a low-cost dense station network is a better choice. These MEMS sensors are cost effective, have a low power consumption and are easy to install. Taiwan’s P-Alert can provide a variety of IMs maps, such as maps of the PGA, PGV and spectral acceleration. In addition to monitoring earthquakes, P-Alert sensors are also used for various seismological studies including fault directional effects and structural health monitoring [100]. A study of P-Alert’s shakemap found that seismic damage is more concentrated in areas with higher values in the PGV distribution map compared to the regional division of the PGA distribution map, and the PGV may reflect seismic damage better than the PGA [101]. Moreover, the applicability of low-cost MEMS sensor-based smartphones in EEWSs has been explored in various countries [102,103,104]. Kong et al. [105] developed the MyShake application by using real-time MEMS from smartphones, whereby they used ML to distinguish between earthquakes and noise, which enabled magnitude estimations and earthquake localizations. The popularity of the MyShake app can be used to reach more areas and people. Furthermore, by combining sensor, cloud computing and network communication technologies, the Internet of Things-based EEWS also holds promise [106]. Alphonsa et al. [107] proposed a simple IoT system that uses an accelerometer connected to a microcontroller to collect and process seismic data and then uses Zigbee to send the data to a receiver connected to a PC that sends alerts to the user.

ML will have an important place in the future of EEWSs, whereby they will provide new solutions for signal identification. Seismic signals are susceptible to local impulse noise from natural or man-made sources, and it is not an easy task to distinguish the true seismic signal from all signals quickly and reliably. Based on the powerful pattern recognition and feature extraction capability of ML, it can be used to improve the accuracy and stability of earthquake early warnings by learning and training with large amounts of earthquake data. ML can also extract important parameters from the raw input data and establish relationships between IMs without considering complex geological conditions, site effects and other factors. This allows ML methods to be widely used in EEWSs [108]. Nevertheless, it should be noted that the interpretability of ML methods is generally weak, which is a limitation. While ML models can be highly efficient at triggering and identifying seismic events and can outperform traditional algorithms, and thereby can reduce false alarm cases in EEWSs [109], their “black box” nature might pose challenges in understanding and explaining the decision-making process.

## 9. Conclusions

After decades of research, EEWSs have undergone significant development. Initially, threshold warning systems were developed, but their warning effect was limited. The PSA was then proposed, which provides a longer TW and formed the basis of current EEWSs in various countries. However, it treats faults as points and has high error rates. To solve this problem, the FF algorithm was introduced, which considers the directionality of faults and predicts the IMs more accurately. Despite these advances, the GMM used by both the PSA and FF algorithm result in large errors due to its empirical nature. To improve the accuracy of the IMs predictions, the physical-based wave-field simulation method was proposed, which incorporates the physical properties of wave propagation. The integrated application of multiple methods can improve seismic information, and the performance of each method needs to be evaluated to determine its accuracy and timeliness. This study found that the PSA can provide the longest TW for distant seismic areas, while the FF algorithm and PLUM method can provide early warnings near the epicenter with more accurate predictions of the IMs’ field distribution. In conclusion, a combination of multiple methods is needed to improve EEWSs, and the evaluation of the performance of each method is crucial to ensure the accuracy and timeliness of IMs predictions. The use of multiple seismic monitoring data and multiple real-time IMs prediction algorithms and models can provide more complete early warning information in different earthquake situations and can help individuals more accurately assess the degree of earthquake hazards and corresponding defensive measures.

## Figures and Tables

**Table 1:** Reviewed articles on different theories of earthquake rupture initiation behavior.

Article	Category	Research Methods	Opinions
[25]	deterministic assumptions	Using extensive global seismic data, measured the period $\tau_{p}^{max}$ of seismic waves and calculated the scalar relationship between *τ*_p_ and *M*w on a log–linear scale.	Information on the final magnitude of the earthquake was available within the first few seconds of the earthquake source rupture.
[26]	deterministic assumptions	The relationship that earthquake rupture initiation behavior has with earthquake magnitude was investigated using the early strong motion records of the near-source *P* and *S* signals, which demonstrated a statistically significant scale.	At the early stage of earthquake rupture, there was a proportional relationship between stress drop and/or active slip surface and seismic moment.
[27]	deterministic assumptions	Analyzed a high-quality seismic database to measure peak displacement (*P*_d_) amplitudes with progressively expanding time windows.	The evolution of *P*_d_ with time was related to the early stages of the rupture process and could be used as an indicator of the final size of the rupture.
[28]	deterministic assumptions	The early *P* wave signals of earthquakes of different magnitudes were analyzed, and an amplitude parameter quantifying the initial peak amplitude was introduced to explore the possible differences in their early rupture.	Small and large earthquakes rupture at different initiation stages, and the final rupture extent of the seismic event was statistically controlled by its initial behavior.
[29]	no correlation assumption	Studied the proportional relationship between *τ*_p_ and *M*w, as well as the effect of this relationship on whether the earthquake rupture was deterministic.	No evidence that the earthquake magnitude could be estimated before the rupture had been completed.
[30]	weak deterministic assumptions	Using a large amount of seismic data, examined how peak absolute vertical displacements evolve over time for different magnitudes.	Small and large ruptures started in indistinguishable ways.
[31]	weak deterministic assumptions	Before the arrival of the *S* wave, the vertical component *P*_d_ measured in the time window was gradually extended and a linear relationship was assumed between log10 (*P*_d_) and the *M*w.	The evolution of *P*_d_ over time suggested a general initial growth pattern that was inconsistent with deterministic models of earthquake rupture.
[32]	weak deterministic assumptions	From a finite fault model database of strong seismic events of magnitude *M*w 7.0–9.0, the average rise time and rupture speeds of each seismic event were analyzed.	They proposed weak determinism, which held that the magnitude of an earthquake could be predicted after it had been nucleated for some time.
[33]	weak deterministic assumptions	Seismic and geodetic data were used to study early rupture indicators to determine if the observations supported deterministic rupture behavior.	Although the initial few seconds were not sufficient to infer the final earthquake magnitude, an accurate estimate could be made before the rupture was complete, which indicated a weak certainty.
[34]	weak deterministic assumptions	The typical temporal rupture behavior of large shallow subduction zone earthquakes was studied using three extensive source–time function catalogs.	The final magnitude could not be accurately predicted until the rupture had developed to a certain size.

**Table 2:** Review of articles on simulated wavefield-based methods for real-time prediction of IMs.

Reference	Methods	Research Methods	Method Performance
[21]	Boundary integral equation	A simple wavefield estimation method that predicted earthquake intensity directly from the real-time seismic intensity observed near the target location.	The method was computationally inexpensive, overcame some disadvantages in terms of point sources and was a powerful method for wavefield estimation that could improve the performance of EEWS.
[82]	Boundary integral equation	Based on Huygens’ principle and the Kirchhoff–Fresnel boundary integral equation, the prediction of subsequent wave fields directly from the observed seismic wave field was proposed.	The method compensated for the shortcomings of the PSA but required a dense observation network; additionally, the warning time was short.
[83]	Radiative transfer theory	A method was proposed to accurately estimate the current wavefield distribution in real time using data assimilation techniques, and then the time evolution of future wavefields was predicted through seismic wave propagation simulations.	The method might mostly reflect the current actual observations, and the assimilation technique minimized the difference between the estimated current state and the actual observations.
[84]	Radiative transfer theory	The path term was incorporated into the numerical shake prediction scheme to predict future wave fields with heterogeneous attenuation structures.	Careful treatment of heterogeneous attenuation structures in numerical shake prediction could help improve ground motion forecasts, especially those with long lead times.
[85]	Radiative transfer theory	A modified Propagation of Local Undamped Motion (PLUM) was proposed by introducing an attenuation factor to the wave propagation.	Improved accuracy and rapidity of seismic intensity distribution compared to the original method.
[86]	Radiative transfer theory	The ALPHA algorithm was proposed; it is based on the Huygens principle, assumes multiple point source models below each observatory and establishes various attenuation relationships to predict intensity.	Compared to existing algorithms, ALPHA enables EEWS to provide accurate warnings to a wider area at an earlier stage.

**Table 3:** Classification of the four types of alarms.

Alarm Category	Abbreviations	Description
True Positive	TP	GM exceeds the threshold and alerts before it arrives
False Positive	FP	GM does not exceed the threshold, but the alarm is issued
True Negatives	TN	GM arrives without exceeding the threshold, and no alarm is issued
False Negative	FN	GM is above the threshold, but no alarm is issued

## Data Availability

Not applicable.
